# Treatment of Hemorrhage from Placenta Previa

**Published:** 1899-01

**Authors:** 


### TREATMENT OF HEMORRHAGE FROM PLACENTA PREVIA.

At the recent meeting of the Obstetrical Society of France, Dr. Sebillotte discussed the treatment of hemorrhage from placenta previa at some length and with much common sense. The proper treatment of this serious obstetrical complication is quite different according to the period of gestation at which it takes place, as well as the variety of the insertion of the placenta and the condition presented by both the mother and the child.

When a hemorrhage, due to a placenta previa, occurs before the sixth month of gestation, it is difficult, if not impossible, to make a diagnosis of its real cause, and the treatment in no way differs from that of a threatened miscarriage, namely, absolute rest in bed, hemostasis and asepsis by vaginal irrigation at a temperature of 48° C., opiates and cold acidulated potions. If miscarriage is prevented, the pregnancy should be watched and all cause of relapse must be avoided.

Is it proper to temporize, by checking the hemorrhage occurring during the latter months of pregnancy? If it takes place in the seventh month, the fetus will have little chance of surviving, and consequently everything must be done to continue pregnancy but bearing in mind that the mother's life is of the first importance. After the seventh month, one of two conditions may be present: the loss of blood is slight, occurring only once, and is easily checked by the means already mentioned, and in such a case nothing will justify the physician to interrupt pregnancy, but he must keep the patient well under his watch. Care should be taken to see that the fetus is in proper position, and if not so, it should be kept in place by an abdominal belt or binder. Everything should be in readiness in case an urgent interference should be indicated, and for this, vaginal antisepsis must be assured, and the patient's general condition kept up by stimulation and tonic treatment, and *each drop of blood lost is to be replaced by a drop of artificial serum.*

When hemorrhage is serious, both on account of its frequency and amount, to temporize is folly. All obstetricians consider the patient's life in danger under these circumstances, as long as she is not delivered of her child. Action is demanded in a triple way, viz.: to arrest the flow of blood, to empty the uterus, and lastly, to combat the anemia and thus improve the general condition. There are a certain number of methods now in vogue, namely, accouchement force, rapid delivery, rupture of the membranes, bi-p lar version, complete detachment of the placenta, and lastly, vaginal tamponade. No matter which of these methods is used, extraction of the fetus should not be delayed as soon as dilation is complete, either by version or with the forceps, and if the fetus is dead, basiotripsy is indicated.

After delivery, the placenta must be removed with care, and if hemorrhage continues, injections of ergotin, hot intra-uterine irrigations, and if necessary, a tamponade, are to be resorted to, without forgetting a general treatment, because at this time all danger has not disappeared.

All the above-mentioned methods have their advantages and their disadvantages, but it is difficult to state just what their respective value may be. But what is most important is the general treatment of the patient.

For slight hemorrhage, rest in bed, hot vaginal irrigations, cold acidulated drinks, and a tonic treatment, are quite enough. For more abundant loss of blood, the horizontal position is not enough, and the blood should be driven to the head and thorax by bandaging the limbs with linen bandages; or even Esmarch's band, while the head should be lowered, and the pelvis and lower limbs are elevated. Syncope is treated with heat and alcoholic frictions.

Hypodermic injections of ether and caffein are excellent stimulators. But most important of all are injections of artificial serum, preferably given subcutaneously. The quantity to be injected each time will vary according to the patient's condition, but usually 200 cc. is enough. According to Pozzi, one injection should not exceed 1,000 cc., and not more than 3,000 cc. should be injected in twenty-four hours. Sebillotte recommends a serum composed simply of seven grammes of chloride of sodium to 1,000 cc. water.—*Annals of Gynecology and Pediatry.*

